# Experiences and practices of traditional healers on snakebite treatment and prevention in rural Malawi

**DOI:** 10.1371/journal.pntd.0011653

**Published:** 2023-10-04

**Authors:** Moses Banda Aron, Manuel Mulwafu, Bright Mailosi, Benno Kreuels, Luckson Dullie, Chiyembekezo Kachimanga, Jörg Blessmann, Enoch Ndarama, Clara Sambani, Fabien Munyaneza, Anat Rosenthal

**Affiliations:** 1 Partners In Health / Abwenzi Pa Za Umoyo, Neno, Malawi; 2 Research Group Snakebite Envenoming, Bernhard Nocht Institute for Tropical Medicine, Hamburg, Germany; 3 Section for Tropical Medicine, I. Department of Medicine, University Medical Center Hamburg, Germany; 4 Neno District Health Office, Ministry of Health, Neno, Malawi; 5 Department of Research, Ministry of Health, Lilongwe, Malawi; 6 Department of Health Policy and Management, Faculty of Health Sciences, Ben-Gurion University of the Negev, Beersheba, Israel; Monash University, AUSTRALIA

## Abstract

Snakebite envenoming remains a public health threat in many tropical countries including Malawi. Traditional healers (THs) have been consulted by victims of snakebites as primary caregivers for millennia. There are no studies in Malawi to understand this phenomenon, therefore, our study aimed to explore the experiences and practices of THs regarding snakebite treatment and prevention in rural Malawi. Between August and September 2022, we conducted semi-structured interviews with 16 THs who were purposefully selected from various locations across Neno District, Malawi. We analysed the interview data using Dedoose software, where we generated codes and grouped them into themes. Out of the 16 THs interviewed, 68.8% (n = 11) were male, and 43.8% were aged between 40 and 60 years. Our study identified five themes: THs’ knowledge of snakes and treatment, the continuum of care they provide, payment procedures, snakebite prevention, and their relationship with health facilities. They claimed a good understanding of the snakes in their area, including the seasons with more snakebites, and were confident in their ability to provide treatment, however, this was not scientifically proven. They offered a comprehensive care package, including diagnosis, first aid, main treatment, and follow-up care to monitor the victim’s condition and adjust treatment as needed. THs provide free treatment for snakebites or use a “pay later” model of service delivery. All THs claimed a “vaccine” for snakebites that could prevent bites or neutralize the venom. However, no formal relationship existed between THs and Health Care Workers (HCWs). We recommend collaboration between HCWs and THs, establishing clear referral pathways for snakebite victims and educating THs on identifying danger signs requiring prompt referral to healthcare facilities.

## Author summary

Snakebite victims in the community continue to seek care from traditional healers for a variety of reasons including cultural influences, socio-economic factors, and the lack of snake antivenom in healthcare facilities among others. Thus, traditional healers play a vital role in the treatment of snakebites. However, one limitation of relying on traditional care is the potential delay or failure of patients to present themselves at hospitals for timely administration of antivenom for those who may require it. We interviewed sixteen traditional healers in Neno, Malawi, to explore their experiences and practices regarding snakebite treatment and prevention. The traditional healers displayed confidence in their treatment methods and offered a comprehensive approach to addressing snakebite cases from diagnosis to medication and follow-up, however, their claims were not scientifically proven. Some traditional healers provided their services free of charge, with some healers requesting payment only when their clients had recovered. Furthermore, traditional healers claimed to possess a snakebite vaccine, and there appeared to be no collaboration or relationship between them and healthcare workers for the management of snakebite cases. We recommend creating spaces for joint learning and collaboration between healthcare workers and traditional healers as they share a common goal of ensuring the recovery of snakebite victims.

## Introduction

Neglected Tropical Diseases (NTDs) are diseases of poverty that impose a devastating human, social and economic burden on more than 1 billion people worldwide, predominantly in tropical and subtropical areas [1–3]. Designated as “neglected”, these diseases afflict the world’s poor; unlike other diseases, they do not receive much attention from the scientific community and funding bodies [4]. Among the 20 NTDs recognized by the World Health Organization (WHO), snakebite envenoming is particularly overlooked despite its significant economic burden on impoverished communities that lack social safety nets in many countries [5,6].

World Health Organization estimates that 5.4 million snakebite cases occur worldwide each year, resulting in 1.8 to 2.7 million cases of envenoming with South East Asia topping the list seconded by Sub-Saharan Africa [7]. Snakebite envenoming is a potentially life-threatening disease caused by the bite of a venomous snake, is responsible for over 100,000 deaths and causes permanent disabilities in more than 400,000 people every year [7,8]. Many snakebite victims in lower and middle-income countries (LMICs) suffer from long-term complications such as deformities, contractures, amputations, visual impairment, renal complications and psychological distress among others [9,10].

Traditional methods, mostly herbal remedies, and concoctions, have been heavily used for snakebite management for generations [11–16]. Studies have also evaluated herbal medicines used in snakebite envenomation and found mixed results in support and against using such herbal medicines [17]. Literature suggests several reasons for the continued use of traditional medicine including transport difficulties, the absence of snake antivenom at health facilities, the affordability of traditional healing methods, and deeply ingrained cultural and traditional beliefs among others [15,16,18,19]. Studies have shown that even if access to medical care were improved, the use of traditional healing would continue in many communities [9,11,15,16,18–21]. Therefore, the integration of THs who rely mainly on medicinal plants and Healthcare Care Workers (HCWs) remains essential in the treatment of snakebites [17].

In Sub-Saharan Africa (SSA), THs are frequently consulted as primary caregivers for snakebite victims [16,18,21,22]. However, a significant number of people continue to die after being bitten by a snake due to severe envenoming, made worse in many cases by delays in obtaining effective medical care at health facilities [15,16]. The delay in seeking medical attention often leads to high mortality rates, even after arriving at a healthcare facility which creates a misconception that conventional biomedical treatments are ineffective [9,11,15,16,18,19,23,24]. Proximity to the patients, quick service, post-healing payment or no payment at all, trust, cure from previous treatments, and perception and explanation of sickness based on the traditional understanding of symptoms are reasons THs are consulted [25]. THs are often approached with the belief that they possess the ability to cure a wide range of ailments including financial prosperity, infertility, and healthcare for diseases such as cancer, mental health conditions, tuberculosis, and more [25–27]. Studies have also suggested that the lack of trust in conventional medicine in Sub-Saharan Africa (SSA) can be traced back to its historical association with colonialism [28–30]. On the other hand, there is very little evidence showing that many of the antivenoms currently used in SSA inclusive of Malawi are suitable or effective [31].

Malawi is home to 66 different snakes of which 11 are medically important [32]. While there are studies done regarding dogs, humans, crocodiles and mosquito bites in Malawi [32–34], studies on snakebites in the country are almost absent as such, prevalence, treatment-seeking behaviour, and patient outcomes are still not known. The only available study done in the Neno district found knowledge gaps among HCWs regarding snakebite treatment. In addition, HCWs reported that snakebite victims go to THs before reporting to a health facility [35]. The use of THs in Malawi is common and has been trusted for generations [26].

While there is limited literature regarding snakebites in Malawi in general, no study has looked at the practices and experiences regarding snakebite treatment and prevention in the country. Studies done in Ghana and Myanmar found that THs are the gateway for snakebite patients, therefore, establishing referral pathways to health facilities for those who first visit the THs remains critical [15,16]. In this study, we assessed THs’ awareness and knowledge regarding common snakes found in their area, common signs and symptoms presented by snakebite victims, the process of treatment of snakebite, ways on how snakebite could be prevented, and how collaboration between THs and HCWs could be established in rural Malawi.

## Methods

### Ethics statement

Institutional support was obtained from the Neno District Health Research Coordinating Committee and Ethical clearance from the Institutional Review Board of Malawi National Health Science Research Committee (NHSRC/21/10/2816; dated 26 November 2021). We also obtained written informed consent from all THs, who either signed or used their fingerprints in case they were illiterate. THs were free to withdraw from the study at any point. We assigned unique numbers to transcripts and recordings and securely stored them on a password-protected computer.

### Study design

We used an exploratory qualitative design of semi-structured interviews with THs to understand practices and experiences on snake and snakebite treatment and prevention. We used semi-structured interviews to gather rich data, uncover new insights and allow for profound conversation with THs’ despite being time-consuming and labour-intensive.

### Study setting

We conducted the study in Neno, Malawi. Malawi, a low-income southern African country bordering Zambia to the west, Tanzania to the northeast and Mozambique forming its southwest, south and southeast borders is home to 18.4 million people as of 2022 [36]. The country covers a geographical area of 118,480 square kilometres. Malawi is divided into three regions, namely: the Southern, Central and Northern regions, with a total of 28 districts. Over 83% of Malawians live in rural areas and 72% of the workforce is employed in the agriculture sector [37,38]. Neno is a rural district in the Southern region of Malawi with approximately 150,000 inhabitants as of 2022 [37]. There are no asphalt roads in many parts of the district, which poses transportation challenges with several areas being almost inaccessible during the rainy season. The district is served by 15 health facilities including 13 primary care facilities and two hospitals, operated and overseen by the Ministry of Health (MOH) and the Christian Health Association of Malawi (CHAM). Seven health facilities (1 hospital and 6 health centres) are located in the western, mountainous part of the district, while 8 health facilities (1 hospital, 4 health centres, and 3 dispensaries) are located on the eastern part with a flat low range area. Neno population is heavily dependent on unindustrialized agriculture which exposes them to snakes [39].

### Study population and sampling

**Fig 1:**
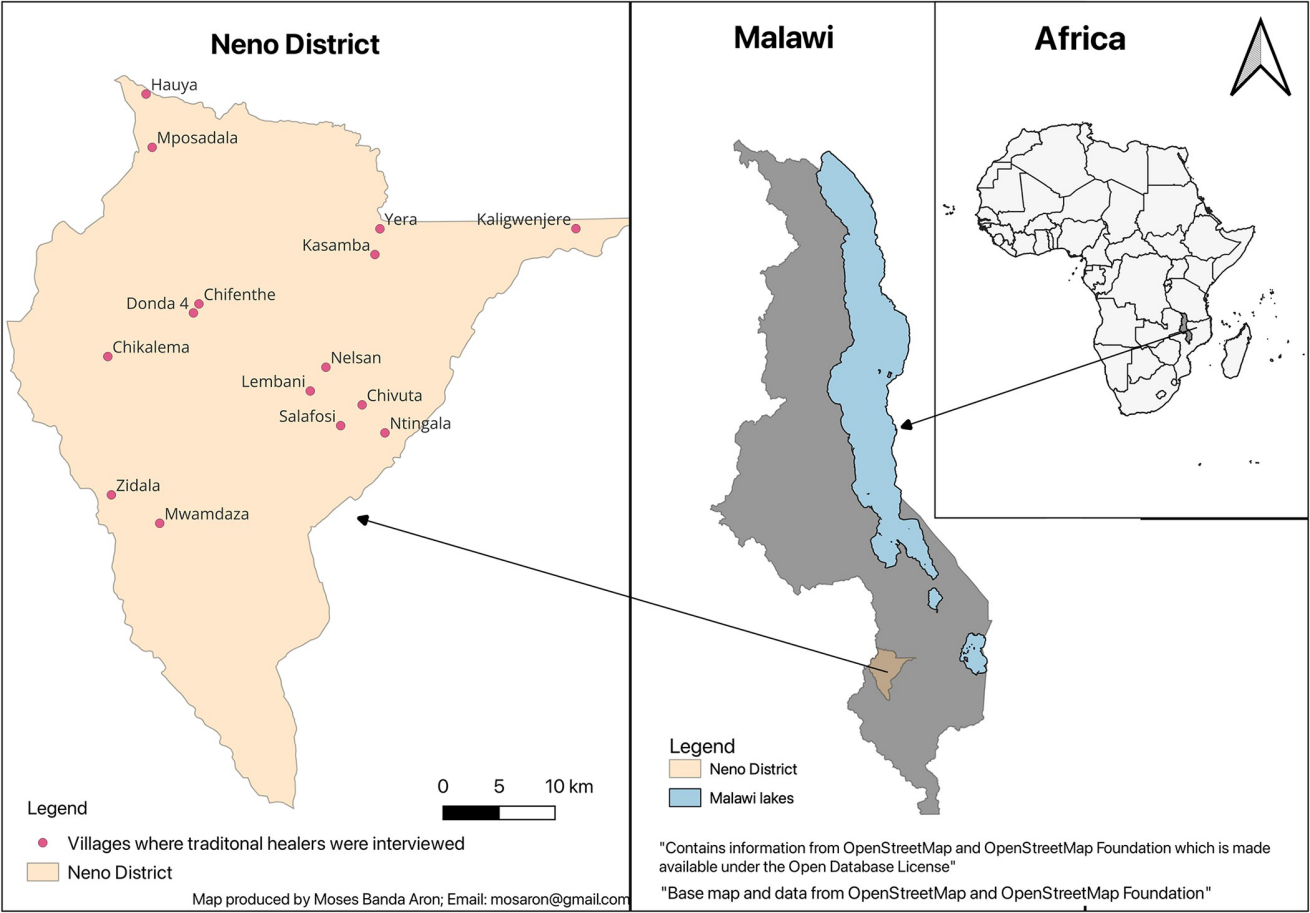
Map of Neno District showing the villages where THs were interviewed.

Our study defined “a traditional healer” as anyone who knows and treats snakebites using traditional medicine. We used purposive sampling to identify THs with the help of Partners in Health’s (PIH) site supervisors who are based at a Health facility supervising Community Health Workers (CHWs) in their catchment areas. Partners In Health, known locally in Malawi as Abwenzi Pa Za Umoyo (APZU), has accompanied the Government of Malawi through the Ministry of Health since 2007 and utilizes CHWs in the delivery of rural community-based primary health care in Neno district [40]. The site supervisors extended the message to CHWs to help in identifying two THs who had treated snakebites in the previous year and also based on the geographical proximity to the health facility. CHW approached the THs face to face-during their monthly household visits and asked if they would be interested in participating in the study. To ensure diverse participation in our study, we deliberately avoided selecting THs from the same or nearby villages as far as possible. Out of the 20 THs approached, 18 agreed to participate while two declined, citing fear due to their lack of registration with the Association of Traditional Healers of Malawi and concerns that the information we were gathering could be used against them. We interviewed 16 of the 18 THs from villages across the district as we could not locate the remaining two at their respective homes despite multiple attempts and could not be reached by phone (Fig 1).

We obtained the “Base map and data from OpenStreetMap and OpenStreetMap Foundation” repository [41] and extracted Africa, Malawi and Neno District boundaries. The map was created using the Free and Open Source Quantum Geographic Information System (QGIS) software [42].

### Data collection

We developed a semi-structured interview guide (S1 Interview Guide) based on the literature and the objectives of the study [9,15,16].

The guide had questions on (1) awareness and knowledge of common snakes found in the area, (2) season and time with more snakebites, (3) signs and symptoms presented by snakebite victims, (4) treatment of snakebite, (5) prevention of snakebites, (6) referral and collaboration between THs and HCWs from the facility. In addition, we collected demographic data including village, age, and sex. The interview guide was initially created in English and subsequently translated into Chichewa, which is the official local language of Malawi and the most spoken language in the Neno District. A back translation was performed to ensure that the questions retained their intended meaning. Following this, the interview guide was tested through a pilot interview with a single traditional healer from the Matandani area in the district, and adjustments were made based on the feedback received. Between 10 August 2022 and 1 September 2022, a member of the study team (B.M.) who has a Bachelor of Science degree and training in Clinical medicine conducted and recorded the interviews at the homes of THs or the practising site in a quiet place following all COVID-19 preventive measures. The shortest interview lasted 20 minutes and the longest was 45 minutes. At the end of the interviews, THs were invited to ask questions and supplement their information with anything relevant to what had been discussed. A second member of the study team (M.B.A.) who has a Master of Public Health reviewed the audio recordings daily and conferred with the interviewer regarding patterns and saturation, which were identified after the 14th interview. Nevertheless, two more interviews were conducted subsequently with female traditional healers to ensure sex dynamics. Furthermore, the interviewer collected some field notes including pictures of medicinal herbs, constituted powders, or poultices used by THs to supplement the collected data.

### Data analysis

We analysed age and sex using Excel and reported counts and percentages. Interviews were verbatim transcribed in Chichewa and then translated into English. However, the transcripts were not returned to participants for comment. Three members of the study team (MBA, AR and MM) each repeatedly read the transcripts, developed a codebook, simultaneously coded sample interviews, and resolved any discordant codes. We analysed the transcripts using thematic analysis as described by Braun and Clarke [43]. The decision was based on our objective to arrive at a broad and rich thematic description of the snakebite problem through the lens of THs without testing a priori hypotheses or theories. Using Dedoose software, we developed an analysis framework iteratively and kept on discussing until a finalised framework was achieved. We compared the codes within and across transcripts and summarised coded extracts grouped into sub-themes and themes as appropriate. We applied the ‘COnsolidated criteria for REporting Qualitative research’ (COREQ) in writing the final report (S1 COREQ Checklist) [44].

### Reflexivity statement

MBA is a Malawian with a background in public health statistics and has been residing in Neno since 2017. MM is another Malawian, specializing in psychology and has worked in Neno for three years. BM, LD, CK, EN, and CS are all Malawian physicians and BM, CK and LD have been living in Neno for over 10 years. BK and JB, are German physicians and have over two decades of experience in studying snakebites in Africa and Asia. FM, a public health expert from Rwanda and living in Neno since 2020, while AR, an Israeli anthropologist, has dedicated more than two decades to conducting research in Malawi. The diverse team, composed of professionals in public health, clinical medicine, anthropology, and psychology, ensured that research was conducted critically and objectively, with a commitment to minimizing personal biases that could influence the outcomes.

## Results

Of the 16 THs who participated in this study, 68.8% (n = 11) were males. Seven (43.8%) were aged between 40–60 years and five (31.2%) were above 60 years old. The demographic characteristics of participants are summarised in Table 1.

**Table 1:** Demographic characteristics of study participants.

Variable	Category	n (%)
Sex	Male	11 (68.8)
	Female	5(31.2)
Age in years	<40	4 (25.0)
	41–60	7(43.8)
	>60	5(31.2)

**Fig 2:**
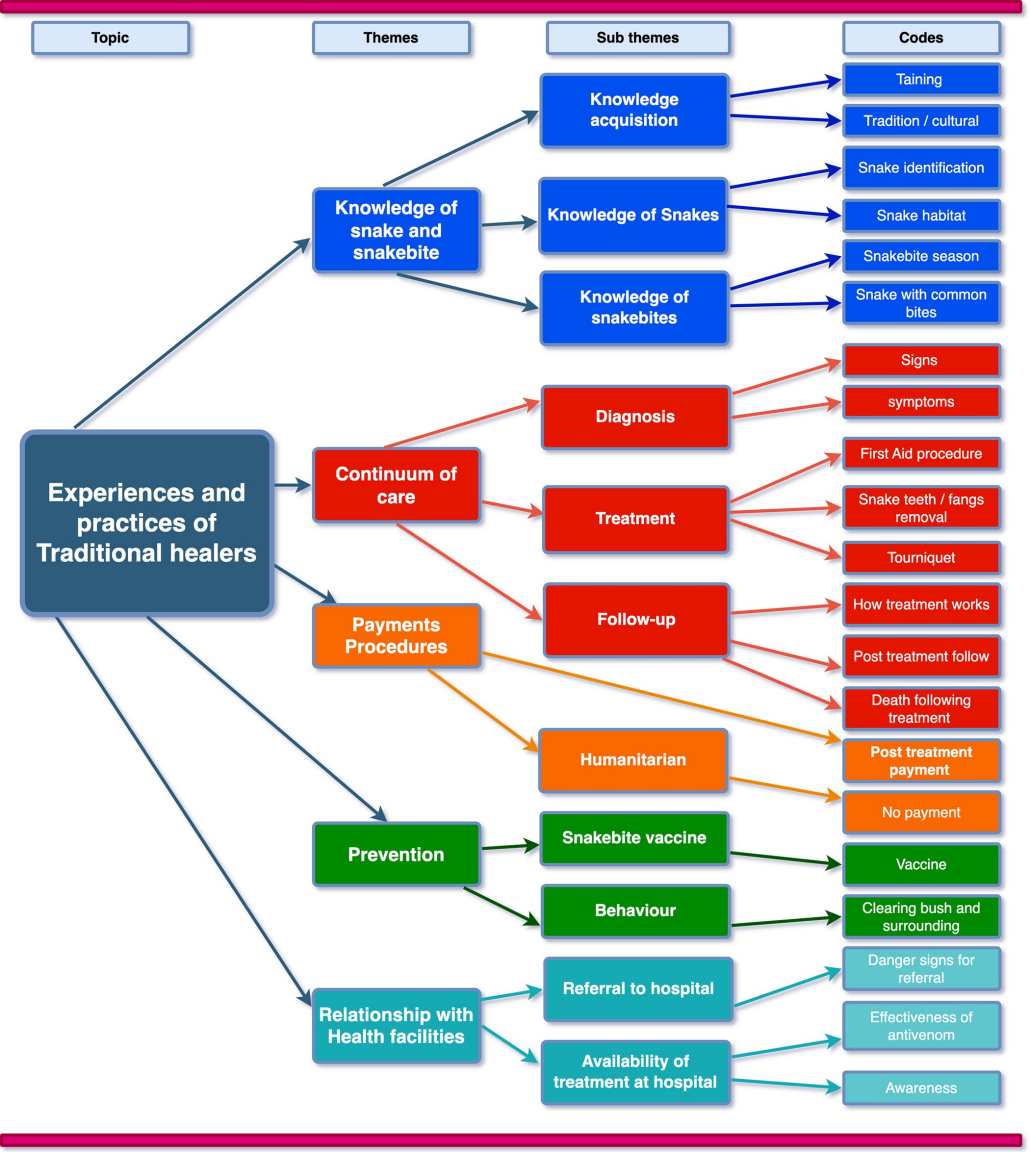
Code tree map for experiences and practices of traditional healers in Neno.

We identified five main themes including 1). Knowledge about snakes and snakebites, 2). Continuum of care, 3). Payment procedures, 4). Snakebite prevention and 5). THs’ relationship with health facilities. Sub-themes and codes derived from the data are shown in the analysis process map (Fig 2).

### Knowledge about snakes and treatment

THs claimed to have a vast knowledge of snakes, their habitat and seasonality, as well as treatment options for snakebites. They were able to describe various snakes including how they could be differentiated, even noting that some species are becoming extinct. Furthermore, THs claimed to know places where snakes could be commonly found in their area, as well as the seasons and times when snakebites were most prevalent as one of the healers explained:

*“…well*, *a puff adder is a beautiful snake and has some mixed colours with square or rectangular boxes like grey*, *brown*, *and other colours depending on where the snake is*. *Puff adders are also short but fatty with a small flat head…”* TH, Zidala village, Magaleta.

Traditional healers’ knowledge was not limited to the identification of snakes, but also extended to an understanding of their habitat and character, as another healer explained:

*“… yes*, *‘Nsalulu’ is one of the fastest snakes and very clever*, *and then there are big snakes like pythons though not common these days*, *I have reserved a forest on that side of my land*, *you can find some of these snakes over there*. *Pythons also come in different forms and sizes*, *there are some small while others can grow up to 2*.*5 meters or even beyond…”* TH, Nelson village, Lisungwi

**Note:** Due to the snake naming challenges that exist in Malawi, “*Nsalulu”* may apply to most *Psammophis* and *Psammophylax* snake families and in some instances as *Naja annulifera*

Furthermore, THs were confident and comfortable with their knowledge regarding treatment and reported that people positively perceived their treatment. One traditional healer reported that he is called by HCWs at the facility to go and attend to a snakebite patient.

“…*Actually*, *they are sent from the hospital to here for treatment*. *A phone call comes; a person has been bitten by a snake and is here at the hospital but is not feeling well; we are trying to assist but he/she is still not getting better*.*…yes*, *they call and I go*, *it is not just a mere person who calls but the hospital doctor himself…”* TH, Kaligwenjere village, Matope

The vast experience and confidence expressed in the interviews form the basis for the treatment they offered to their patients.

### Continuum of care

THs offer a full package of care for the snakebite victim. The treatment is systematic and organized with stages from diagnosis, treatment and follow-ups. THs diagnose the victim based on the signs and symptoms including swelling of the bitten part, sweating, and shortness of breath among others. In addition, they also probe the victim or their relatives about the incident:

*“…sometimes people are bitten by something else and come claiming snakebites*. *For example*, *certain scorpions are venomous*. *So normally a person will present with some small marking on the spot where it had bitten*, *and it will leave teeth*. *If bitten by a puff adder*, *the marks are larger and some blisters will develop after some minutes around the mark*. *They are some that do sweat a lot including the bitten part*, *their eyes change to red and have difficulties in breathing and the saliva dries or they feel as if they don’t have saliva in their mouth…”* TH, Zidala village, Magareta.

In their interviews, THs also shared their diagnosis routines:

*“…Aah*, *I normally ask where*, *and they say here*. *When the snake bites*, *it leaves teeth*, *and in those teeth thus where the venom is and it is the venom that will rush and go to the heart*. *Some come with a swollen leg*, *feel pain and the heart beat fast…”* TH, Chivuta village, Zalewa

Before the actual treatment is offered, all THs interviewed give first aid to the snakebite victims which includes medicine for pain relief, one that causes a person to vomit to reduce the effects of snake venom and remove snake teeth or fangs:

*“…As I explained*, *all this is done with urgency*, *I don’t know exactly what type of snake in case the person does not say*, *therefore*, *I give pain relief medicine and also that the person vomits or rushes to the toilet to reduce the power which otherwise goes to the heart*. *That is part of first aid for me…”* TH, Nelson village, Lisungwi

Of the 16 THs, only one mentioned the application of a tourniquet as a first-aid procedure:

*“…At first*, *I start by giving the person oral medicine to vomit as a snakebite leaves poison in the person’s body*. *And then you see that the bitten area is darkening*, *there I don’t take long to tie here with a string*, *and then the person vomits and defecates from there I can give ‘Mphini’…”* TH, Ngwenyama village, Luwani

Note: Mphini is a traditional method that involves creating small incisions on the skin using a razor blade, with the purpose of introducing powdered or poultice herbal medicine into the body. In the long run, small tattoos are formed, often appearing in pairs.

The main snakebite treatment given by THs includes various medicinal herbs that are constituted and applied through *“Mphini”*. THs also built the trust of the people by applying the medicine to themselves first before giving it to the snakebite victim, as one of the healers explained:

*“… It is a traditional medicine from trees and herbs*, *for example*, *this tree shrub*, *locally called “Bwazi”*. *I scratch the stem and take a few leaves*, *put them in a cup*, *and stir then it produces a foam*. *I then take a bit before giving it to the victim so that in case a person dies*, *they should not say that I gave him/her poison*. *Then we have another herb locally called “Dululu” which is for teeth removal and healing*, *I make a poultice and then apply it on the bitten part and also give it through “Mphini” as a snakebite vaccine…”* TH, Mwamdaza village, Magareta

**Fig 3:**
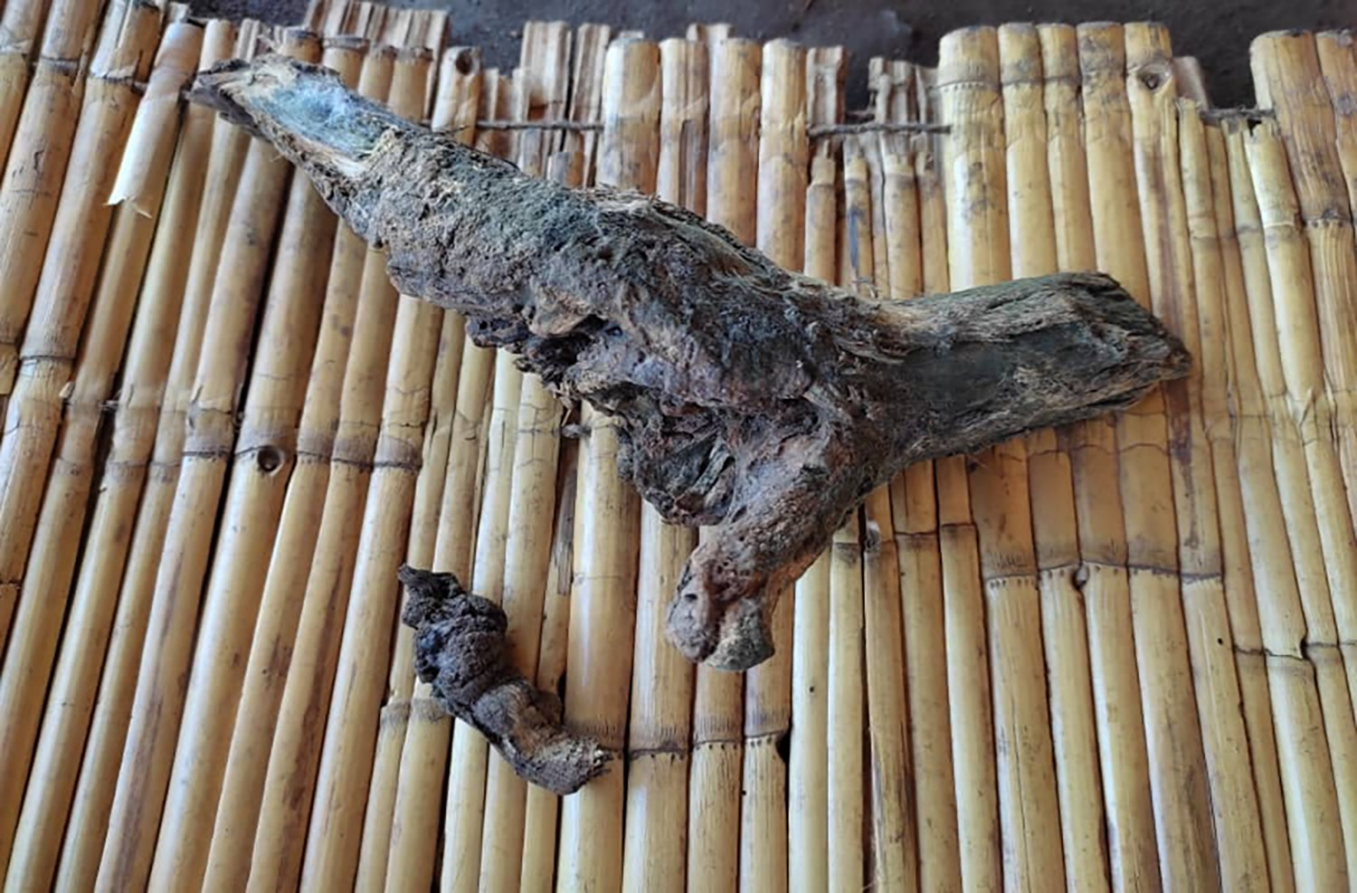
Dried stems of Dululu (bigger one) and Bwazi (small one) used as a treatment.

**Note:** Bwazi and Dululu are local herbs used by traditional healers for snakebite treatment (Fig 3).

All THs interviewed described a follow-up procedure after the snakebite treatment is complete to check on how their client is healing, and if there are any complications. These follow-ups could also include additional treatment or a referral to the hospital. The follow-up procedure was addressed in the interviews as part of the continuum of care:

*“…I visit them*, *I visit them in their homes and greet them and also check on them*, *the next day or the other you find they come on their own walking*, *they say the leg is okay and I can walk on my own as you can see*. *For us herbalists and the hospital*, *there is no big difference…”* TH, Salafosi village, Zalewa

The importance of follow-up on patients was also addressed as interviewees addressed their responsibilities:

*“…I visit them to see how they are healing*. *After 3 days I go and see them*, *how are you doing*? *And they respond ‘Today*, *I am doing very well’*. *If not*, *I apply a different medicine*. *Why should we just give the medicine through ‘Mphini’ without following them up*?…*”* TH, Yela village, Midzemba

Throughout the study, no death of a snakebite victim was reported by any of the THs following their treatment. However, one traditional healer reported that the victim fainted in the process of treatment, and transport was arranged for the person to be referred to the hospital, luckily, after some minutes the patient recovered:

*“…No*, *nothing like that [death] has happened before but I remember*, *one fainted while I was assisting her but did not die*. *At that hour*, *the transport was already there to take her to the health facility*, *however*, *after some minutes*, *she was okay and demanded porridge as she was feeling hungry*. *We provided the porridge and she did not go to the hospital*. *After two days*, *I followed up and found that she was doing okay and able to walk…”* TH, Mwamdaza village, Magareta

Furthermore, THs associated death with neglect on the part of the patient and delays in seeking care:

*“…No*, *for the ones that I have ever assisted*, *it has never come to that accident you are talking about*. *But if the person is careless without vaccination and delays having the medicine and the pain gets worse can die*, *but when they hurry to the traditional healers for assistance then the person cannot die…”* TH, Yela village, Midzemba

From diagnosis to follow-up and referral, THs were meticulous in describing the continuum of care and the risk associated with delays in care.

### Payment procedure

All THs use a “pay later” model of service delivery to snakebite clients. While some expressed their responsibility to treat people for humanity and never ask for any money as they consider snakebite as an accident, act of witchcraft, or some misfortune, others charge a payment for their services based on the socio-economic status of the person or the village, as one traditional healer explained focusing on the humanitarian aspect:

*“…I just do it for humanity*, *it’s up to them to come and say thank you in their own will*. *What I do is just assist the person knowing that it was an accident*. *If the person gets better*, *I am happy and it is up to them to come and say ‘thank you’*, *so I don’t even mention it*. *However*, *as per our tradition of thanking one another*, *they come and give any amount they have*…*”* TH, Chikalema, Neno Parish

Another interviewee focused on the socio-economic considerations involved in the process of payment for her services:

“…*According to the socio-economics of our village*, *most people are poor as I am as well*, *they just give a small amount*, *I normally*, *tell them whatever*, *they have*. *Some give 2000 kwacha (USD2*.*00) while others give 7000 kwachas (USD7*.*00) considering that we also go and look for these in the bush*…” TH, Chivuta village, Zalewa

While all THs saw themselves as professional service providers, their payment approach was deeply rooted in their relationships with community members.

### Snakebite prevention

All THs interviewed in the study saw themselves as providing prevention services from clearing the bush, applying snake repellent by spraying the grounded powder around their house or surrounding, or planting the medicine herbs around their house. These environmental prevention measures were explained in the interviews:

*“…Okay*, *here we just take care of our surrounding*, *clearing bushes so that it is clear*. *Again*, *I take some medicine and spread it inside or around the house so that it turns back when the snake smells the medicine*. *This medicine is a plant called “bwazi”*, *we prepare and sprinkle it around and when the snake smells it does not come any close*. *If it was inside the house*, *it goes away by itself…”* TH, Yela village, Midzemba

The THs’ role in planting medicinal plans was also addressed:

*“…We dig the roots of a certain tree*, *this tree when we dig*, *we grind in a mortar and then spread the medicine around the home sometimes we plant the seeds of the tree if we have them*. *From there we sprinkle it inside the house or just put it inside the house*, *and the snake cannot enter…”* TH, Salafosi village, Zalewa

In addition, all the study’s THs claimed they had a “Vaccine” for snakebite. The vaccine can be given to someone to prevent being bitten by snakes or if accidentally bitten, assuring that the bite is harmless. According to the THs, the vaccine is applied through “Mphini” and can be given at any age even to children, and will remain active in a person’s lifetime:

*“…I give the medicine which vaccinates one from snakebite through ‘Mphini’*. *For example*, *a small child like one over there*, *if you vaccinate him*, *the snake will never bite him or if bitten*, *the venom will not work on him*. *You will find him walking on the same day and wonder*, *is it the same person*? *Thus*, *if he was vaccinated against snakebite*…*”* TH, Chivuta village, Zalewa

**Fig 4:**
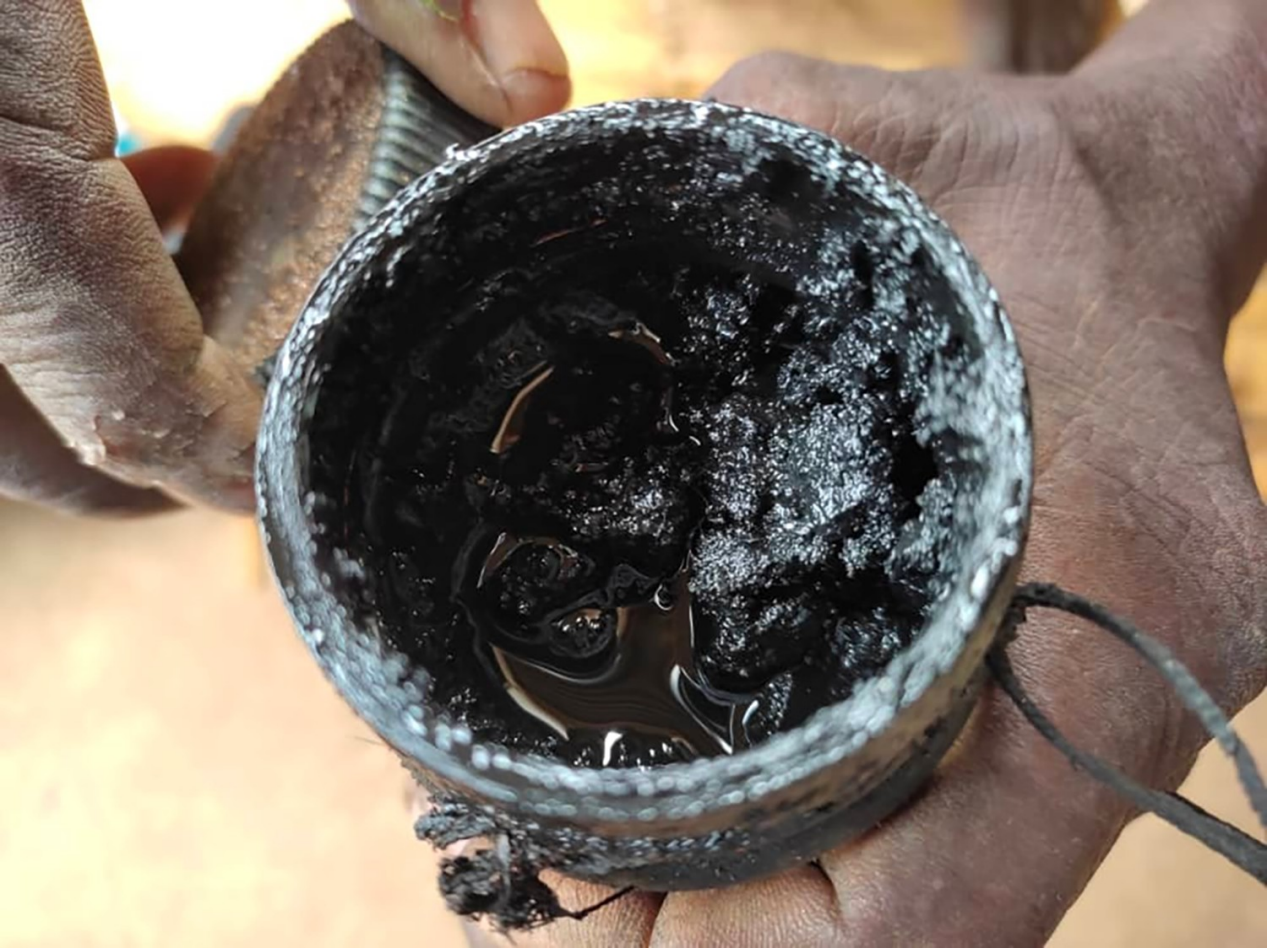
Snakebite vaccine made of a mixture of heads of snakes and herbs.

Several healers described the vaccine as prepared purely from herbs, while others use a mixture of the herbs and heads of the snakes (Fig 4)–they cut snake heads and dry them up and mix them:

*“…I mix different kinds of heads of snakes*, *every kind of snake found in our area*, *a puff adder*, *cut off its head*, *mambas do the same*, *“Mbuvi”*, *equally as well as boomslang and then dry them off*. *I combine them with other stems–about five trees we get them in the bushes*, *but I don’t burn them*, *just dry them completely*. *I take a mortar and pestle and start grinding to powder*. *I then put them in a clean bottle*, *get used engine oil*, *add*, *and then stir*. *I cover it and leave it in the house*. *Anyone willing to get this vaccine through ‘Mphini’ would be protected for life*. *No matter what*, *even if you come across a snake*, *it cannot bite you*, *the snake surely will run away…”* TH, Donda 4, Neno district

**Note:** Due to the snake naming challenges that exist in Malawi, this local name applies to most *Lycodonomorphus* and *Natriciteres* snake families

Snakebite prevention measures offered by THs were understood as both environmental and individual protecting the home, garden, and individual body.

### Relationship with health facilities

Our study identified the complex relationship between THs and the biomedical health system. In their interviews, THs described part relationships with health workers and even previous instances of professional forums of THs and HCWs. Some healers noted that they had more interaction with healthcare providers when they worked as traditional birth attendants, while others were involved in messaging campaigns during the onset of the COVID-19 pandemic, both to promote prevention and to learn how they could protect themselves from the disease. THs expressed a desire for a stronger collaboration on snakebite treatment, just as they had done for other diseases:

*“…I was a traditional birth attendant*, *we used to go to Lisungwi before Zalewa had a health facility*. *They used to give us cotton plus other tools for that work… as traditional birth attendants’ work ended due to the risk of HIV and also that women can die*, *there has been no interaction with the hospital and so even for the snakebites…”* TH, Chivuta village, Zalewa

The same sentiment addressing the loss of the relationship with health workers in the context of the COVID-19 pandemic:

*“… ah*, *currently*, *there is no relationship*. *I would be happy if we could have one*, *I remember at the onset of COVID-19*, *healthcare workers came here with masks*, *gloves*, *and hand sanitisers for my work*, *of course*, *this was not about snakebite*. *I was very happy to receive those items as they protected me from my work…”* TH, Nelson village, Lisungwi

The idea of a formalized space for collaboration with health workers was also discussed in the interviews:

*“… as traditional healers*, *we used to have interface meetings with the health care providers so that they know what exactly we work on and what they do*, *but after our president died*, *we no longer do that*. *The health care workers should tell us what we should be doing*, *for example*, *giving us some signs that we can be observed in our patient that can qualify for a straight-away referral or if it is something we can try from this end*…*”* TH, Kasamba village, Midzemba

Alongside the desire for more collaboration with the health system, THs had a strong mixed reaction to the snakebite treatment at the health facility. While some acknowledged that healthcare facilities do offer treatment for snakebites and recommended that victims who live near such facilities should seek treatment there directly, others expressed doubts about the effectiveness of treatment offered at such facilities. One traditional healer even reported that healthcare facilities could not treat snakebites and that seeking treatment there could result in disability for a patient who would otherwise have recovered fully:

*“…I know that the health facility has medication for snakebites for example when you go there*, *they will give you an injection or some fluids which I believe treat snakebites*. *I think it depends on the area*, *some are very close to the health facility*, *my recommendation would be that they go directly to the hospital but maybe those very far like here*, *they should get assistance*. *I have never heard that someone has died at the hospital due to snakebite*, *which means they can also assist…”* TH, Zidala village, Magareta.

THs’ criticism of hospital care was often expressed with regard to patients and their health outcomes:

*“…what they do at the hospital is just to add the patient’s pain*. *I feel so sorry for that*. *My other grandchild was bitten by a snake near Neno Church of Central African Presbyterian Church*, *she stayed in the hospital for so long and never thought she would put shoes again on her feet*. *She was discharged from the hospital with a very big wound at first*. *When they came here*, *I challenged them that if a week goes without this wound getting better*, *ask for other better help*, *but it went well*…*”* TH, Chifenthe village Neno DH

The complexity of the relationship between THs and health facilities was woven into what could be understood as conflicting expectations. On one hand, there is a professional criticism of the facilities’ ability to provide quality care for snakebite victims, while at the same time a desire to collaborate and create shared spaces for mutual learning.

## Discussion

Studies have shown that THs play an important role in the care of snakebite victims [11–16,18,21]. However, in the Malawian context, not much is known about their professional knowledge, practices of care, and experiences in snakebite treatment. To the best of our knowledge, this is the first study conducted in Malawi on the knowledge, experiences, and practices of THs regarding snakebite treatment and prevention. We found that THs have considerable knowledge when it comes to the types of snakes prevalent in their area and the seasons during which more snakebites occur. THs offer a comprehensive treatment package that includes diagnosis through victims’ signs and symptoms, first aid, administering treatment, and following up with the victim post-treatment. The majority of THs participating in this study were using a pay-later model for their services while others were providing treatment free of charge. The THs’ interviews claimed to have vaccines that could either prevent snakebites or prevent adverse effects if someone was bitten. Furthermore, while it has been shown that health workers in facilities know traditional practices [35], we found that there was a weak relationship between THs and healthcare facilities, with most THs acknowledging the need to establish a stronger relationship.

Our study found that THs claimed to be knowledgeable of the snakes found in the area, the seasons with more bites, and very confident regarding the treatment they offer. However, their claims were not scientifically proven compared to HCWs as stated in a previous study in Neno [35]. The confidence that THs have in their treatment was understood as building trust among the people they served. It is not surprising that studies conducted in Myanmar and Ghana have reported that even if health facility treatment were to improve, snakebite victims would still seek treatment from THs because their treatment is perceived to be good and trustworthy [15,16]. The traditional healers in this study offer a continuum of care for snakebite victims from diagnosis to follow-up. Unlike in other settings where “black stone” has been used as a treatment [11,16,19,45–47], such was not reported among the interviewed THs in Neno. We found that THs use herbs plants and a blend made of a mixture of the head of the dead snake and herbs to treat snakebite. Moreover, THs reported that they provide follow-up care to their patients, which is known to positively impact social connectedness and support for fellow community members [15]. Our study found that THs claimed a successful snakebite treatment rate with no reported deaths. However, this could not be verified as no records were available unlike at the hospital. In addition, it is difficult to ascertain the number of snakebite victims treated by traditional healers. The high treatment success rate claimed could be attributed to dry bites and bites from non-venomous snakes available in the district as described by the THs. Nonetheless, the success rate builds trust in the community and reinforces the preference for seeking care from THs, as reported in studies conducted in Cameroon, Eswatini, Myanmar, and Rwanda [11,15,18,19,48]. Additional research is needed in Neno to gain insights into the perspectives of actual snakebite victims on the treatment process provided by THs, validate traditional healers’ knowledge and also look into establishing a THs treatment record register.

THs’ “pay later” model of service delivery and humanitarian treatment is deeply rooted in the community and has an impact on health-seeking behaviour. Although biomedical health services are considered "free" in Malawi, studies have shown that patients still have to bear the expenses of transportation, hospitalization, and medication purchase in case of stockouts at health facilities [49,50]. Given the socio-economic background of individuals in rural areas such as Neno and their proximity to THs in the event of a snakebite, trusting THs could be the most practical and affordable solution. Similar findings were reported in Kenya and in Ghana where snakebite victims preferred THs over health facilities as they would accept payments at a later date while others provided the treatment for free [16,45]. A participatory action research study conducted in Myanmar revealed that even with improved health facility services, individuals who have been bitten by snakes would still prefer traditional medicine due to its low-cost [15]. Further research is required to understand this phenomenon, its underlying mechanisms, and potential implications for healthcare delivery and patient outcomes in rural settings in Malawi.

In Neno, THs plant herbs that act as "snake repellent" around their homes and sprinkle them inside their houses for prevention. Similar approaches have been used by people in Benin, Ethiopia, Zimbabwe, Uganda, and Oman [51–55]. However, studies investigating the approach of repellents are scarce and there is no evidence for the effectiveness of these approaches. In addition, THs in Neno claimed that they have a snakebite “vaccine” made from a mixture of herbs and heads of snakes which can protect one for life or if accidentally bitten, assure that the bite is harmless. A similar combination of medicine preparation has been reported in studies done in Botswana, Eswatini, India, Kenya, Nicaragua, and Tanzania for treatment and prevention [18,56–59]. Pharmacological analysis studies are required to ascertain the chemical composition and antivenin properties of concoctions made by THs in Neno District, Malawi.

In the context of the broader health community in the district, our study found a weak and somewhat undefined relationship between THs and healthcare facilities when it comes to snakebite treatment, despite the shared goal of promoting patient recovery and well-being. The presence of both support and opposition to collaboration between THs and HCWs, as observed in our study, was anticipated. Unlike HCWs, who undergo standardized medical training, the training of traditional healers is intricate and diverse. Some traditional healers acquire their knowledge through generational teachings, while others attribute their expertise to spiritual influences which may be against biomedicine. Therefore, there is not uniformity in their perspectives on the relationship with medical institutions. Similar findings were reported in a synthesized review aimed at enhancing snakebite care in rural settings [60]. The treatment of snakebites in health facilities was recommended by some THs, while others expressed doubts about the efficacy of such treatment. This has serious implications, particularly as snakebite victims typically seek assistance from THs first. Studies done in Rwanda, Cameroon, and Myanmar among snakebite victims and THs reported doubt about the hospital treatment effectiveness [11,15,19]. THs in Neno strongly recommend a collaborative approach with HCWs to ensure that they are educated about danger signs that guarantee a straight referral of snakebite victims to the hospital. There is extensive literature on the integration of THs and modern medicine for various diseases and conditions, snakebites could equally benefit [61,62]. Further research is needed to investigate how THs in Neno can be integrated into the healthcare system, particularly in the management of snakebites. One possible approach could involve providing THs with training and community registers to document the cases they treat. This would complement the data gathered by health facilities and help strengthen the overall healthcare system.

Our study has some limitations. Firstly, the interviews were conducted in Chichewa, transcribed, and then translated into English, which may have compromised the quality of the data. Nonetheless, we took measures to ensure the accuracy of the translations by back-translating four transcripts to ensure that their meaning was not lost. Secondly, our research was confined to THs in Neno exclusively, and the perspectives we gathered may be influenced by area-specific cultural mannerisms. Nevertheless, we believe that the experiences and practices of THs in rural Malawi and other similar rural settings could be comparable.

Overall, traditional healers were confident in their snake knowledge and snakebite treatment, however, these were not scientifically proven. THs provided their services on humanitarian grounds with others using the “pay later” model of service delivery. In addition, THs claimed to have a snakebite vaccine that would protect one for life and similarly, this was not proven scientifically to be effective. And finally, there was no relationship between THs and the HCWs. We recommend that HCWs and THs collaborate and learn from each other, rather than disregarding the value of THs who have gained the trust of the community. One viable solution is to create a forum where THs can interface with HCWs, as seen during the onset of the COVID-19 pandemic and traditional birth attendants’ work. Furthermore, it is crucial to establish clear referral pathways for snakebite victims, involving both THs and HCWs. To ensure that referrals are made promptly and appropriately, traditional healers need to be educated on recognizing danger signs that require immediate referral to healthcare facilities. Further research is required to assess the perception of the community toward THs for snakebite treatment

## Supporting information

S1 Interview Guide Semi-structured interview guide for Traditional healers. (DOCX)

S1 COREQ Checklist COnsolidated criteria for REporting Qualitative research’ (COREQ) Checklist. (PDF)
