# Supplementary material for: Experiences and practices of traditional healers on snakebite treatment and prevention in rural Malawi
Source: PLoS Negl Trop Dis. 2023 Oct 4;17(10):e0011653. doi: 10.1371/journal.pntd.0011653 (PMC10550111; doi:10.1371/journal.pntd.0011653)
Supplement: S1 Interview Guide — (DOCX) [file pntd.0011653.s001.docx]

**S1: Semi-structured interview guide for Traditional healers**

**Demographics**

1. Catchment area _____________________
2. Village__________________________
3. Age of traditional healer (years) ____________
4. Sex _____________

**Main Questions**

1. What are the common snakes found in your area and their living and hiding places?
2. Give brief descriptions of the snakes identified as responsible for the bites of your victims. Probe on Venomous vs non-venomous
3. Can you describe what those snakes look like? At what times do people get bitten?
4. What time of year and what time of day or night are these snakes likely to be out and active?
5. What are some of the common symptoms shown by the snakebite victims who report at your treatment centre?
6. How do you treat snakebite victims? Probe for more, like a mix of concoctions, etc. take a picture if possible.
7. How much does it cost to treat snakebites?
8. Describe briefly your treatment procedure.
9. What is the best method to prevent snakebites around houses?
10. Are you aware of the first aid measures for a snakebite patient?
11. Have you ever given first aid treatment to any patient with snakebite in your area? If so, how do you manage a patient?
12. How do you advise patients after you treated them?
13. Do you advise them to go to a health facility?
14. If yes, when and which health facility?
15. Are there any clinical signs that make you decide to refer a snakebite victim to a healthcare facility?
16. Do you follow up on patients?
17. Have you ever lost [the death of] a patient during treatment? What happened?
18. How is your relationship with your area's healthcare facilities (hospitals)?
    1. What perceptions do you think the people working at the hospital have about your treatment methods and approach to snakebite?
    2. What perceptions do you think the community members have about your treatment methods and approach to snakebite?
19. Could something be improved in the collaboration between you (the traditional healer) and the healthcare facilities?
20. How?
21. Do you get updated about the availability of antivenom at local hospitals and pharmacies? Where do you get that information from?
22. Is antivenom effective at treating snakebites?
23. How could referrals from you to the hospital be made easier?
24. Have you heard of treatments at the hospital that you would like to be able to perform yourself?
